# Exploring LLM-powered multi-session human-robot interactions with university students

**DOI:** 10.3389/frobt.2025.1585589

**Published:** 2025-06-03

**Authors:** Mauliana Mauliana, Ashita Ashok, Daniela Czernochowski, Karsten Berns

**Affiliations:** ^1^ Robotics Research Lab, RPTU Kaiserslautern-Landau, Department of Computer Science, Kaiserslautern, Germany; ^2^ Center for Cognitive Science, RPTU Kaiserslautern-Landau, Department of Social Science, Kaiserslautern, Germany

**Keywords:** social robots, human-robot interaction, large-language models, generative AI, user studies

## Abstract

This exploratory study investigates how open-domain, multi-session interactions with a large language model (LLM)-powered social humanoid robot (SHR), EMAH, affect user perceptions and willingness for adoption in a university setting. Thirteen students (5 female, 8 male) engaged with EMAH across four weekly sessions, utilizing a compact open-source LLM (Flan-T5-Large) to facilitate multi-turn conversations. Mixed-method measures were employed, including subjective ratings, behavioral observations, and conversational analyses. Results revealed that perceptions of robot’s sociability, agency, and engagement remained stable over time, with engagement sustained despite repeated exposure. While perceived animacy increased with familiarity, disturbance ratings did not significantly decline, suggesting enhanced lifelikeness of SHR without reducing discomfort. Observational data showed a mid-study drop in conversation length and turn-taking, corresponding with technical challenges such as slower response generation and speech recognition errors. Although prior experience with robots weakly correlated with rapport, it did not significantly predict adoption willingness. Overall, the findings highlight the potential for LLM-powered robots to maintain open-domain interactions over time, but also underscore the need for improving technical robustness, adapting conversation strategies by personalization, and managing user expectations to foster long-term social engagement. This work provides actionable insights for advancing humanoid robot deployment in educational environments.

## 1 Introduction

As human-robot interaction (HRI) increasingly explores language-enabled social robots, large language models (LLMs) have emerged as powerful tools for building interactive, intelligent systems (Williams et al., 2024). Among the four broad areas of LLM-powered robotics, communication is the primary focus of HRI researchers, which can be divided into language generation and language understanding (Kim Y. et al., 2024). Language generation for social humanoid robots (SHR) can be task-dependent, supporting goal-specific dialogue, or task-independent, enabling open-domain conversations for social engagement. Open-domain dialogue systems are expected to (1) comprehend user response semantics, (2) generate coherent responses that fit the conversation history and match a predefined persona and style, and (3) engage users emotionally (Huang et al., 2020). While LLMs excel in language understanding and generation, they lack the ability to engage in real-time, embodied interactions within physical environments. LLM-powered agents address this gap by enabling multi-turn reasoning, planning, and generalization through language understanding (Xi et al., 2025).

In HRI studies, LLM-powered SHRs primarily use large pre-trained language models that enable zero-shot or few-shot learning without updating model weights, relying on proprietary models like GPT-3.5 and GPT-4, or open-source models like Llama-2 and Vicuna (Williams et al., 2024; Kim Y. et al., 2024; Xi et al., 2025). In addition to their out-of-the-box capabilities, fine-tuning LLMs such as OpenAI’s GPT-3, BERT, Google’s PaLM, and T5 for specific HRI tasks has also been shown to enhance real-time robot communication by better mimicking human conversational patterns and behavioral cues, likely due to large-scale pre-training on human-generated text (Zhang and Soh, 2023). However, LLM-powered robots present two major challenges: hallucination, where the robot deviates from the intended context, and high computational overheads, which may hinder offline deployment on embedded systems such as SHRs with limited processing power and system memory. Due to the opaque and sometimes inconsistent nature of LLM outputs, caution is advised when integrating them into social robots (Ranisch and Haltaufderheide, 2025). Nevertheless, similar to human-driven solutions often used in HRI, such as wizards or expert demonstrators, LLMs can still serve as valuable, human-like components despite their imperfections Williams et al. (2024). Previous studies have shown that physically embodied SHRs consistently demonstrate higher engagement compared to virtual agents, particularly in relationship-oriented tasks where physical presence enhances social engagement and rapport (Nishio et al., 2021). Building on this, recent work has demonstrated that repeated interactions with SHR can further strengthen user perceptions, emotional disclosure, and wellbeing over time, highlighting the importance of studying relational change across multiple sessions (Laban et al., 2024). Combining physical embodiment with advanced LLM capabilities and repeated interactions offers a promising strategy for a more natural human-robot relationship.

This work extends from a prior study that introduced ChitT5, a custom fine-tuned conversational model based on Flan-T5, designed for small-talk (open-domain) in HRIs (Ashok et al., 2024a). Evaluated with 22 participants in a first-time interaction (ice-breaker) session, results showed strong correlations between rapport and perceived conversational competence, though users still saw the robot as a “stranger”. Despite promising engagement, limitations of the developed robot dialog system included a lack of factual accuracy, robot emotion handling, and conversational memory, steering a need for an improved system deployed in a long-term multi-session setting. Although recent studies in HRI with SHRs have explored emotion, memory, or personalization individually, the present work integrates all three into a single, locally deployed system to support natural, multi-session interactions. Simultaneously achieving the expected goals of open-domain systems remains challenging due to the complexity of multi-turn conversational reasoning and the lack of standardized methods for evaluating dialogue quality (Huang et al., 2020). While technical evaluations of the fine-tuned model were performed, we argue that in the context of a highly anthropomorphic robot such as Ameca (placed closest to human-like appearance and behavior in the poster Research, Social and Entertainment Humanoids^1^), real-world user perceptions provide a more meaningful measure of system success, as embodiment fundamentally shapes user expectations.

**FIGURE 1:**
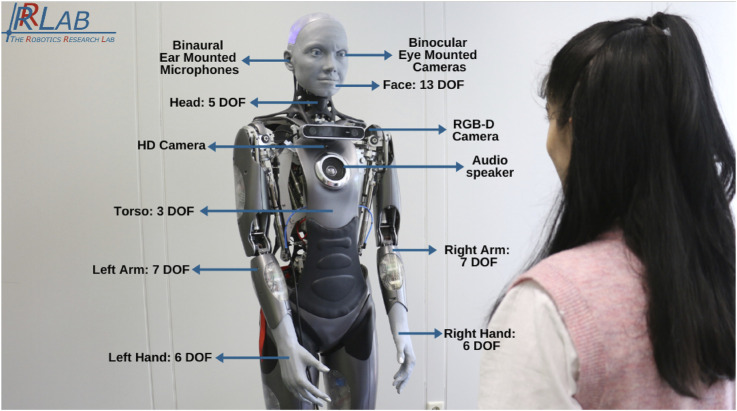
EMAH (Empathic Mechanized Anthropomorphic Humanoid) robotic system implemented on Gen 1 Ameca robot from Engineered Arts.

In this study, we built a custom LLM with under a billion parameters into a dialogue system for the SHR Ameca robot, evaluated over four interaction sessions (see Figure 1). The dialogue system was customized using a pre-trained Flan-T5-Large model (Chung et al., 2024), fine-tuned on a downstream task using daily conversation datasets, and combined with retrieval-augmented generation (RAG) methods. We then integrated this system into the SHR Ameca’s robotic framework developed at the robotics research lab (RRLab), named the EMAH system (here on referred to as EMAH) (Ashok et al., 2024b). To evaluate the developed open-domain dialog system, a controlled lab experiment was conducted with 13 university students participating in four interaction sessions.

Exploratory Question: How does open-domain interaction across multiple sessions with an LLM-powered social humanoid robot affect user perceptions and willingness to adopt it as a companion?

A demo recording of the first and second interactions of the robot with the author is available here^2^.

## 2 Related work

LLMs are emerging as quick prototyping tools in HRI, enabling full interaction pipelines for SHRs similar to earlier Wizard-of-Oz methods (Williams et al., 2024). For example, Nadine, a gynoid robot utilizing GPT-4 with prompt engineering through the ReAct framework, demonstrated advanced conversational abilities and real-time emotion generation based on the ALMA affect model (Kang et al., 2024). Similar to our multi-module system, this work highlights efforts to combine language generation, persona consistency, and emotion modeling. The use of LLMs to generate fitting robot emotions paired with dialogue is an area of social robotics that continues to grow (Mishra et al., 2023; Sievers and Russwinkel, 2024). Earlier studies have explored embedding non-verbal cues, such as gestures and facial expressions on QTrobot, within task-independent language generation to foster greater user engagement and empathy, using fine-tuned GPT-3 models to support the interactions (Khoo et al., 2023). Despite advances in emotional expressiveness, conversations with LLM-powered robots often remain superficial due to limited memory and context retention (Irfan et al., 2023; Ashok et al., 2024a). To address this, a key strategy has been to provide conversational history as context within the system prompt to generate a more coherent conversational flow. SHR platforms such as Furhat (Irfan et al., 2023), Pepper and Nao (Billing et al., 2023), QTrobot (Khoo et al., 2023), and Nadine (Kang et al., 2024) have been equipped with proprietary GPT models to enhance their social capabilities. However, most existing work relies on external APIs or cloud-based solutions, limiting transparency and reproducibility. Therefore, to ensure transparency, reproducibility, and control, the open-source model deployed in this work was fine-tuned on publicly available conversation datasets, deployed locally, and its weights are publicly available.

User perception of an SHR’s sociability and agency plays a critical role in determining a user’s willingness to use or adopt it. However, these perceptions are not shaped solely by system capabilities but are deeply influenced by the SHR’s physical and social design. Users tend to naturally treat SHRs as social actors, expecting them to behave in human-like ways and build social relationships over time (Forlizzi and DiSalvo, 2006). Studies have shown that while appearance can initially attract users, sustaining meaningful engagement requires more sophisticated social behaviors, such as expressing emotions, maintaining memory-based personalization, using gestures, facial expressions, and demonstrating empathy (Cho and Nam, 2023). Failure to meet these behavioral expectations can lead to disappointment, emphasizing the mismatch between user expectations and robot capabilities, creating an expectation gap which plays a decisive role in user evaluation and acceptance of social robots (Rosén et al., 2022). A prior study focusing on robot emotions across three advanced humanoids, including Ameca, attributed its realistic facial expressions to the uncanny valley phenomenon (Berns and Ashok, 2024).

Building on these previous findings, our study addresses several gaps. While Nadine (Kang et al., 2024) and QTrobot (Khoo et al., 2023) demonstrated emotional generation and engagement techniques, they did not systematically measure how user perceptions evolve across multiple sessions. Similarly, studies highlighting expectation gaps (Rosén et al., 2022) and physical embodiment effects (Forlizzi and DiSalvo, 2006; Berns and Ashok, 2024) have emphasized the importance of managing user expectations but often relied on single-session evaluations. Following the recent focus on long-term relational development with robots (Laban et al., 2024), we adopt a multi-session approach to investigate how repeated open-domain interactions with an LLM-powered SHR affect user perceptions and willingness to adopt the robot as a university companion. To this end, we examine.

• RQ1: How does repeated interaction with an LLM-powered social humanoid robot influence user perceptions of sociability, agency, and engagement?
• RQ2: How does prior experience with robots relate to developing rapport and perceived companionship over multiple sessions?
• RQ3: How does user familiarity with the robot affect perceptions of disturbance or comfort over time?
• RQ4: How are user expectations associated with their willingness to adopt the robot as a social companion?

## 3 Methods: Designed LLM

The system is designed using a compact LLM, fine-tuned for multi-task, which serves as the core of the robot’s dialogue system. A prompting mechanism is utilized to personalize the inference process and create a robot persona, enhancing the user interaction experience (Whittaker et al., 2021). A retrieval-augmented generation (RAG) approach (Lewis et al., 2020) is integrated to mitigate hallucinations and improve factual accuracy, where additional data is sourced explicitly from the RRLab website. The system also involves emotion recognition grounded in Plutchik’s eight basic emotions model (Plutchik, 1989), enabling real-time emotional recognition in the HRI loop. The system detects human emotions from their utterances and uses this information as an additional context to generate the robot’s responses. Then the emotion of the generated robot response is also analyzed, and utilized as input to the robot’s expression control system. The source code for the model and the chatbot interface demo is available here^3^.

The dialogue system consists of four main components: a Google API-powered speech recognition module for converting speech to text, a fine-tuned language model, a memory management system for storing and retrieving information, and the Finroc framework. Finroc is a robotic integration platform that manages communication with robots, text-to-speech capabilities, and physical behaviors with finite-state machines implemented as a module (Reichardt et al., 2012). The dialogue system supports multi-session interactions by distinguishing between new and returning users, enabling long-term companionship through personal information detection, retrieval, and memory management. Additionally, at the end of the conversation session, a summarizer module is used to conclude the conversation into a short story, which, along with the conversation history, is stored as the robot’s memory of the user.

### 3.1 Multi-session interaction module

The multi-session interaction module consists of two main components: the conversation agent, the brain that manages the conversation process, and the robot communication system, which oversees system communication, sensors, and the robot’s actuation. The conversation agent is developed using Python, while the robot communication system utilizes the Finroc Framework, which is written in C++. A Pybind wrapper enables the conversational agent to interface with the robot system by allowing Python to call C++ functions.

**FIGURE 2:**
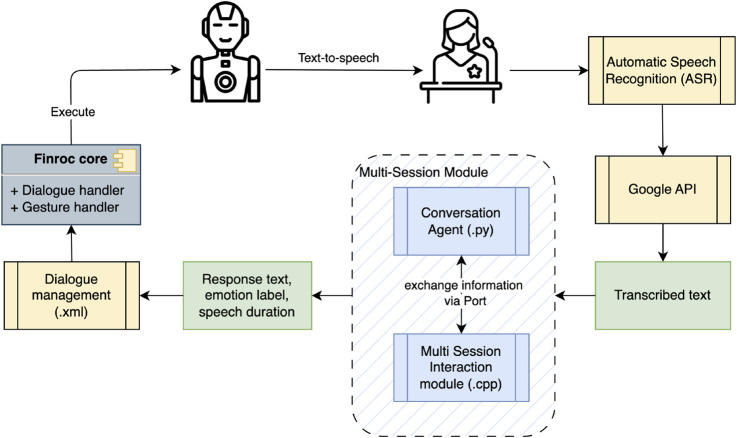
Workflow of designed dialogue system on human-robot interaction.

The system flow is illustrated in Figure 2. Based on the generated text and emotion label sent by the multi-session module, the robot’s speech, facial expressions, and gestures are managed through a modular system operating within the Finroc framework. A finite-state machine (FSM) method, whose structure is initialized from an XML configuration, is employed for dialogue management control. Utilizing a pre-existing module developed and stored in the Finroc library, the emotion label generated by the conversational agent can be linked to activating the corresponding facial expressions and robot gestures based on the defined labels. A default text-to-speech (British English, Lucy from Acapela group^4^) allows the robot to vocalize the generated text.

### 3.2 The conversation agent design

The conversation agent is designed with the aim of making the robot able to engage in natural, human-like interactions. Using a multi-task fine-tuned LLM as a core, the system is expected to be capable of engaging in light conversation with humans, including daily chit-chat, awareness of human feelings, demonstrating empathy, and providing some facts about the RRLab research group.

#### 3.2.1 Datasets

Four publicly available English datasets are used to fine-tune the main multi-task model: DailyDialog (Li et al., 2017), EmpatheticDialogues (Rashkin et al., 2018), Topical-Chat (Gopalakrishnan et al., 2023), and BlendedSkillTalk (Smith et al., 2020). Additionally, the SamSun datasets (Gliwa et al., 2019) are employed to fine-tune the summarizer model, while a handcrafted dataset containing all relevant information about RRLab is created for the retrieval feature.

The combined dataset is preprocessed and formatted into three distinct tasks.

1. Response Generation: An autoregressive, context-aware text generation task. The input includes past and current utterances along with the emotion label of the current utterance. The target is the next utterance.
2. Emotion Classification: A multi-label classification task where the input is an utterance and the target is one of Plutchik’s eight emotion labels.
3. Background Information Detection: A binary classification task where the input is an utterance and the target is a “Yes” or “No” label indicating whether background information is present.

Fine-tuning is a transfer learning approach that modifies source model parameters to learn target tasks in specific domains. The best performance of multi-task learning can be obtained with balanced sharing from each task (Upadhyay et al., 2024). Therefore, data augmentation was carried out to reduce the dataset size gap among tasks by increasing label data.

#### 3.2.2 Data preprocessing

The following sections provide a detailed explanation of each type of dataset preprocessing.

##### 3.2.2.1 Response generation

     An emotionally intelligent social robot is a crucial aspect of our design system. To be a pleasant companion, a robot should partially understand human emotions through speech, appearance, and behavior. Additionally, the robot needs to respond with polite sentences and perform gestures based on the perceived emotion (Szabóová et al., 2020). In large language generative models, emotional stimuli can be injected by either adding text that represents a specific emotion (Li et al., 2023) or by directly stating the type of emotion (Mishra et al., 2023).

For the response generation task, the DailyDialog, Topical-Chat, and EmpatheticDialogue datasets are used. However, since the emotion label of the text is also needed as context in the input, along with the previous utterance, we utilized a helper tool, namely, the emotion classification model, to provide the emotion label. Additionally, this tool was also used to standardize the emotion label based on Plutchik’s eight-emotion model.

##### 3.2.2.2 Emotion classification

**FIGURE 3:**
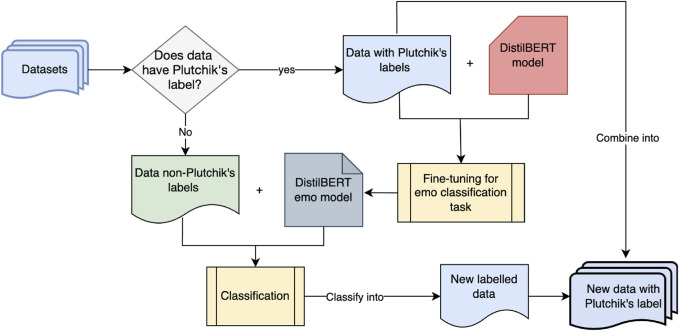
Emotion classifier fine-tuning and data augmentation to Plutchik’s label process.

     With the most labeled emotion categories, the EmpatheticDialogue datasets were selected as the baseline dataset to fine-tune a helper model for an emotion classification tool. The emotions were mapped to Plutchik’s labels beforehand. The mapping of EmpatheticDialogue’s emotions into Plutchik’s eight basic emotions is listed in Table 1. Once the mapping was complete, the emotion classifier model was created by fine-tuning the DistilBERT-Base model with this data. The model fine-tuning and data augmentation process is illustrated in Figure 3.

**TABLE 1:** Mapping of EmpatheticDialogue dataset emotion label into Plutchik’s basic emotion label.

Plutchik’s label	EmpatheticDialogue label
Anticipating	Anticipating, Anxious
Joy	Joyful, Content
Trust	Trusting
Fear	Afraid, Terrified
Surprise	Surprise
Sadness	Sad, Lonely, Devastating
Disgust	Disgusted
Anger	Angry, Annoyed, Furious

##### 3.2.2.3 Background information detection

     The BlendedSkillTalk is a conversational dataset with human annotations on four categories: knowledge, empathy, personal situations, and personal background (Smith et al., 2020). For a background information detection task, the BlendedSkillTalk data with personal background annotations was used as the main dataset. However, since the dataset contains only 7,017 entries, more synthesized data was needed to reduce the imbalance with other tasks. A binary classifier for background information was therefore created by fine-tuning the DistilBERT-base model on the available data as a supporting tool. The Topical-Chat dataset was then augmented as an additional source by labeling data using the trained classifier. Before labeling, the data was filtered based on the “knowledge source” attribute, and only entries labeled as ‘FS’ and “personal knowledge” were included.

As the final preprocessing step, a profanity check^5^ with a 60% threshold was applied to reduce the likelihood of the final model 167 generating inappropriate words. The final number of data pairs for each task is presented in Table 2.

**TABLE 2:** The structure and size of the dataset used for multi-task fine-tuning.

Task	Datasets	Total pairs
Response generation	DailyDialog, Topical-Chat, EmpatheticDialogue	179,511
Emotion classification	DailyDialog, EmpatheticDialogue	104,304
Background information detection	BlendedSkillTalk, Topical-Chat	98,398

All datasets were then encapsulated into a prompting template for the Seq2Seq input format as presented in Table 3.

**TABLE 3:** Dataset format based on task type for multi-task fine-tuning model.

Task	Prompt template	Example
Response generation	Current utterance emotions are a {emotion}. By considering the emotion, predict the next response: \ n {history}	input_text =“““Current utterance emotions are a “Joy”. By considering the emotion, predict the next response S1: Are you going to the annual party? I can give you a ride if you need one S2: Thanks a lot. That’s the favor I was going to ask you for.””” target_text = “The pleasure is mine.”
Emotion classification	Please predict the Plutchik’s emotion label for this utterance: \ n {text}	input_text =“““Please predict the Plutchik’s emotion label for this utterance I used to scare for darkness””” target_text = “fear”
Background Information detection	Please predict if this utterance contains personal background information: \ n {text}	input_text =“““Please predict if this utterance contains personal background information I used to scare for darkness””” target_text = “yes”

##### 3.2.2.4 Summarizer

     The SamSun datasets^6^ was used to fine-tune the summarization model. This dataset contains 16,000 messenger-like conversations, each accompanied by a summary of the corresponding dialogue (Gliwa et al., 2019). The dataset was structured such that the conversation history serves as the input while the summary is designated as the target.

##### 3.2.2.5 Information retrieval

     The information for RRLab was gathered by scraping articles from the RRLab website and its internal repository. Each article or piece of information was saved in an individual TXT file, resulting in a total of 54 articles. This data was then converted into vector embeddings using the BAAI general embedding (BGE) model (Xiao et al., 2023) and stored in the Chroma database (Chroma, 2024) as non-parametric data. The decision to use the BGE model was made solely after inspecting three leading embedding models for semantic retrieval functionality. The three candidate models were Sentence Transformers^7^, Instructions Embedding^8^, and the BGE model. The inspection follows the same procedure for all embedding models, which are placed in the same system designed to run the same embedding and searching tests. Based on the inference time, computational complexity, and output produced, BGE was chosen. This evaluation result was subjective and may not be applicable in different cases since it is only a self-evaluation process.

**FIGURE 4:**
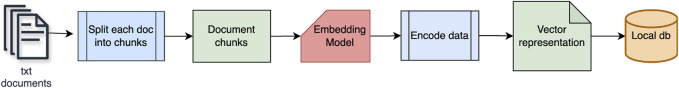
Embedding local information workflow.

     The workflow of transforming data from a repository of documents into a vector embedding representation saved in the Chroma database is illustrated in Figure 4. During this process, the RecursiveCharacterTextSplitter chunking strategy from the LangChain Framework was applied, with a window size of 1,000 and an overlap size of 100.

#### 3.2.3 Model fine-tuning

A study conducted by Kim C. Y. et al. (2024) found that robots powered by LLM exhibit distinct preferences for various tasks, which are influenced by the unique characteristics of each task. Referring to this finding, we fine-tuned four models to create a complete workflow for a multi-session HRI scenario. The fine-tuning process is executed using Trainer, a function within the HuggingFace API library, in PyTorch.

##### 3.2.3.1 Response generation (multi-task) model

     A multi-task model was developed by fine-tuning a version of the Flan-T5-Large language model, optimized for three specific tasks: response generation, emotion classification, and background information detection. Also, to prevent the loss of previous information from the fine-tuning process, the low-rank adapter (LoRA) (Hu et al., 2021) technique was applied.

     Training configuration: epochs 20, batch size 32, learning rate 1e-3, warmup ratio 0.01, weight decay 0.01, optimizer AdamW.

     LoRA configuration: rank 16, alpha 32, dropout 0.05, and task type SEQ_2_SEQ_LM.

##### 3.2.3.2 Emotion classification model

     The emotion classification model was fine-tuned from the DistilBERT-Base model for a multi-label classification task. This model was created as a helper tool for augmenting emotion-label data.

     Training configuration: epochs 10, batch size 64, learning rate 2e-5, weight decay 0.01, callback early stopping with patience 2 and threshold 1.0, and evaluation strategy IntervalStrategy.STEPS.

##### 3.2.3.3 Background information detection model

     The background information detection model was fine-tuned from the DistilBERT-Base model for a binary classification task, using “yes” and “no” as labels. Similar to the emotion classification model, this model was developed to help increase the size of the labeled background information dataset.

     Training configuration: epochs 10, batch size 64, learning rate 2e-5, weight decay 0.01, callback early stopping with patience 2 and threshold 1.0, and evaluation strategy IntervalStrategy.STEPS.

##### 3.2.3.4 Summarizer model

     The summarizer model was a fine-tuned Flan-T5-Large model specifically created for text generation tasks. This model functions exclusively as a summarizer, supporting the design of the conversational agent for multi-session scenarios.

     Training configuration: epochs 10, batch size 8, learning rate 1e-3, warmup ratio 0.01, optimizer AdamW.

     LoRA configuration: rank 16, alpha 32, dropout 0.05, and task type SEQ_2_SEQ_LM.

#### 3.2.4 Model evaluation

The evaluation metrics used for the fine-tuned models varied depending on the specific task. For classification tasks such as the emotion (multi-label) classification model and the background information (binary) classification model, performance was assessed using accuracy, precision, recall, and F1-score. In contrast, models designed for text generation tasks, including the multi-task response generation and summarizer models, were evaluated using BLEU, METEOR, and ROUGE-L metrics. This evaluation was conducted during the fine-tuning phase, where various hyperparameters were adjusted to optimize model performance. The final model used for inference achieved scores as detailed in Table 4 for the classification task and Table 5 for the text generation task.

**TABLE 4:** DistilBERT fine-tuning model evaluation score.

Task type	Accuracy	Precision	Recall	F1
Emotion Classification Model	0.78	0.77	0.77	0.77
Background Information Detection Model	0.66	0.66	0.66	0.66

**TABLE 5:** Flan-T5 fine-tuning model evaluation score.

Task type	BLEU	METEOR	ROUGE-L
Response Generation (Multi-task) Model	0.023	0.24	0.40
Summarizer Model	0.136	0.39	0.41

It is important to note that the evaluation metric scores used during the fine-tuning of the Flan-T5 model provide only a general indication of learning progress and do not fully reflect the model’s real-time inference performance across all tasks (Zhang et al., 2024).

#### 3.2.5 Retrieval-augmented generation (RAG)

Internal information about the RRLab was embedded into the robot system using the RAG approach. Lewis et al. (2020) introduced RAG to mitigate the hallucination effect in LLMs by incorporating a retrieval function to access external knowledge (non-parametric data), thus providing additional information for the LLM during text generation. This enhancement aimed to improve the accuracy and reliability of the generated results.

This work implemented the RAG concept using a question-answering tool within the LangChain Framework. The LLM utilized was a multi-task fine-tuned model, with the non-parametric data source being the RRLab internal information data. In real-time retrieval, the vector embedding data stored in Chroma was accessed via the RetrievalQA function, which is a vector retrieval tool within the LangChain framework, specifically designed for question-answering purposes (LangChain, 2024).

### 3.3 Real-time conversation system flow

The multi-turn conversation system was equipped with person identification to support a long-term interaction scenario. At the beginning of the interaction, the robot would ask the speaking partner to mention their username. According to the name detected, the system will identify the person as a new encounter or revisited before starting the conversation flow.

**FIGURE 5:**
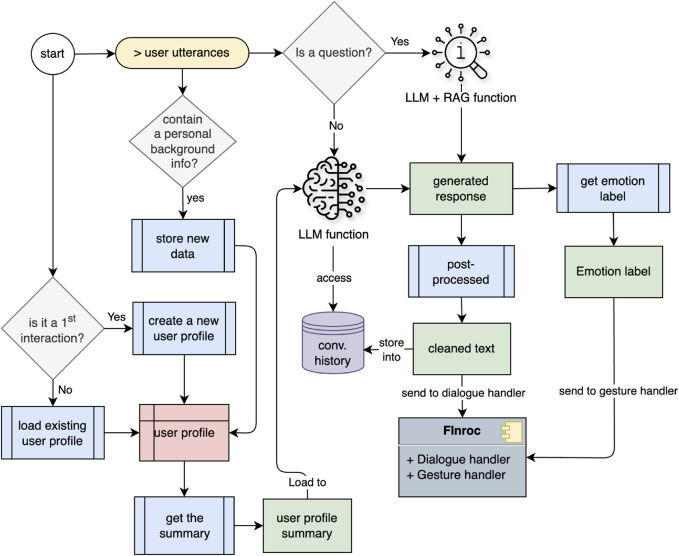
Conversation workflow of Multi-session Interaction Module within the Finroc framework.

One of the limitations observed in the preceding study (Ashok et al., 2024a) was that in some cases, such as when the interlocutor responded with a short answer, the model repeated the same sentence from the previous turn. Therefore, to tackle this problem, we implemented a minimum quality control in the current work by utilizing a rule-based approach and a simple machine learning method to obtain better response sentences. The conversation flow in the system is illustrated in Figure 5. Additionally, the main process of the multi-session interaction module is presented as pseudocode in the supplementary material section “Real-time Conversation Algorithm”.

Two approaches were used to generate responses: RAG + LLM and LLM functionality. A gradient-boosting classifier from the NLTK library analyzes sentence structure. When a question is detected in the transcribed text, we assumed the interlocutor may expect factual information. Consequently, we activate the RAG + LLM function; otherwise, the LLM function is always triggered. As previously mentioned, during real-time interactions, the model detects not only emotions from the spoken sentences but also whether the utterances contain personal background information. At the end of each interaction, the conversation history (including human utterance and robot response), the emotion detected for each utterance, and a list of background information identified from the human utterances are stored as a user profile in the form of a JSON file.

**FIGURE 6:**
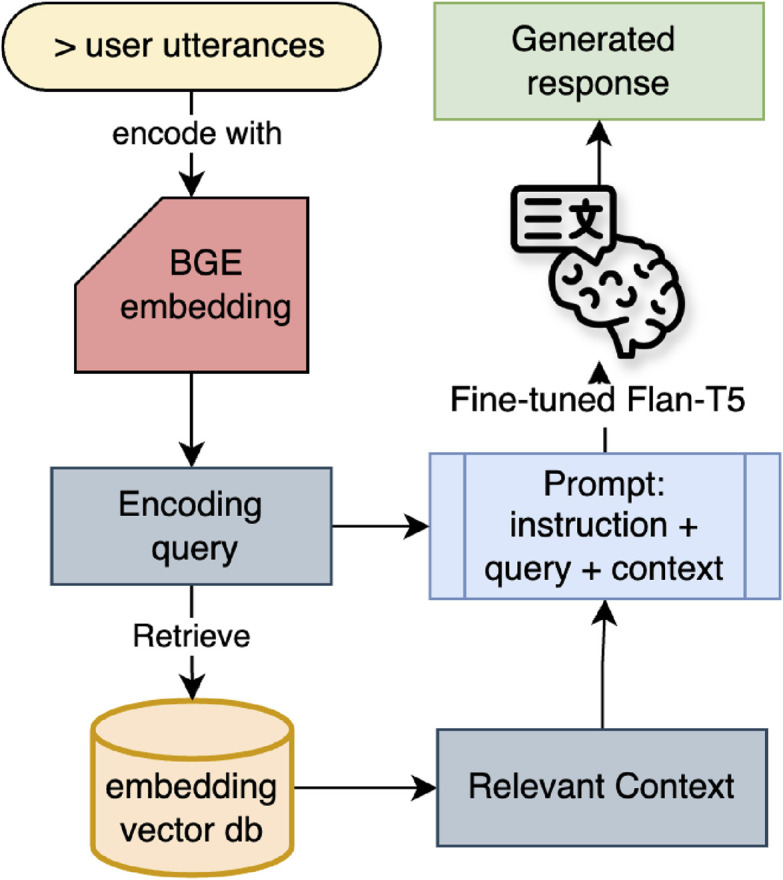
Response generation using RAG approach.

1. RAG + LLM Functionality. The retrieval feature in this function extracts relevant context from the user’s query using a pre-created vector database. Subsequently, the user’s query and the retrieved information are combined in a prompt template and sent to the language model to generate a response. The RAG approach emphasizes factual information and does not consider human emotions when generating the response. This functionality is referred to as RAG + LLM, representing the process of generating a response with RAG and then utilizing a multi-task LLM to obtain the emotion label from the generated text. The flow of response generation on this path is shown in Figure 6
2. LLM Functionality. The LLM function aims to create a friendly conversational tone. It considers both the context from the previous utterance turn and the emotional aspect of human interaction when generating responses. In addition, a robot persona was appended to the prompt template during the inference process to personalize the robot as a student companion. A cosine similarity check with a threshold of 0.6 was applied to prevent the generation of identical responses. If the current generated text failed this check, the response was re-generated by lowering the top-p value of the sampling method in the inference hyperparameter to 0.80.

**FIGURE 7:**
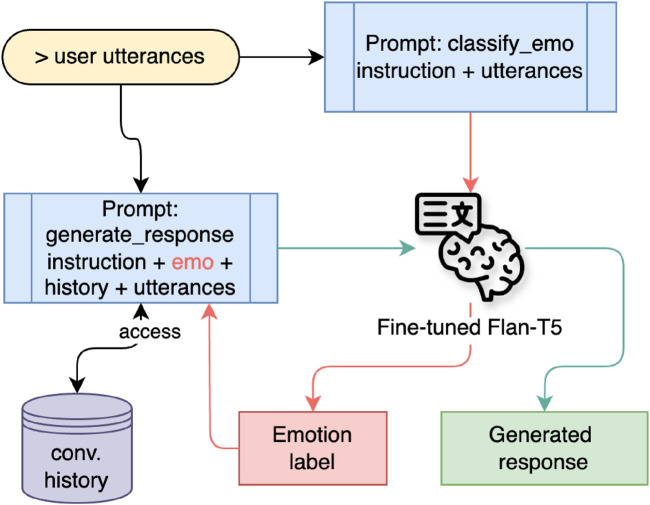
Response generation using LLM functionality.

     The default hyperparameter settings in the inference process are num_beams = 5, max_new_tokens = 90, top_p = 0.90, top_k = 150, repetition_penalty = 1.15, early_stopping = True, do_sample = True. These hyperparameter configurations were determined after several experiments on the parameter values, particularly regarding the number of beams, top-p, and top-k. The flow of response generation with LLM functionality is illustrated in Figure 7.

### 3.4 Conversation summarizer and robot memory

A conversation summarizer function serves as a complementary module to support multi-session interactions. From a technical standpoint, this module aims to reduce real-time buffer memory in the system by storing less data across multiple conversation sessions. From a cognitive perspective, it enhances naturalness by mimicking the human-like ability to recollect past conversations as well as imperfections through forgetfulness. A study by Biswas and Murray (2015) revealed that incorporating cognitive imperfections, such as forgetfulness, successfully fosters initial attachment bonds with humans. Considering humans’ tendency to imperfectly recall past events, particularly conversation content, we hypothesized that providing only summary or partial information to the robot in subsequent interaction sessions could simulate human-like forgetfulness.

In the HRI process, the summarization function ran separately after each interaction session. The conversation history, which served as the robot’s episodic long-term memory, was stored in a JSON file. This file was then passed to the summarization module, which generated a new JSON file containing the summary appended to the end of the conversation history. The robot system structured memory based on personal encounters. Each JSON file represented one user profile. When encountering a new person, the system created a new JSON file, while in subsequent meetings, new information was appended to the existing user profile.

## 4 Multi-session student and robot interaction

### 4.1 Experiment design

A controlled experiment was conducted at RPTU Kaiserslautern-Landau, Germany. The participants were recruited through a multi-channel approach, including posters in university public spaces, the university mailing list system, and the experimenter’s social media. The eligibility criteria required participants to be university students aged between 19 and 35, proficient in English, and willing to commit to attending four interaction sessions with the EMAH robot scheduled in April 2024. Each participant was allocated specific time slots to interact weekly with a robot throughout four sessions. The experimenter gives a short monologue about the procedure and scenario at the beginning of each session and asks for the participant’s consent to undergo the experiment. The participants were also required to complete the questionnaire every session before and after the interaction session. In addition, at the fourth session, besides the questionnaire, at the end of the interaction, the experimenter will also interview the participant to get an objective perspective of the interaction experienced by the participant. No technical information is disclosed during the experiment session to reduce bias; only the recorded data from participants’ interactions is shared. However, all technical details about the system are explained after the participant completes the experiment.

### 4.2 Participants

The HRI experiment initially involved 15 participants, but two withdrew during the interaction sessions. Consequently, 13 participants (5 females and 8 males, with no non-binary or diverse individuals) successfully completed all four sessions. Their ages ranged from 21 to 32 years old. The majority of participants (seven in total) were from computer science. Two were from cognitive science, two from Embedded Systems, one from Commercial Vehicle Technology, and one from Physics. In the initial interaction session regarding contact with robots, data were collected from all 13 participants. Five indicated prior experience with a university robot before their involvement in the experiment, while six reported no prior interaction. The remaining two participants chose not to disclose their experiences. A similar distribution of responses was observed regarding participants’ familiarity with personal assistant systems such as Siri, Alexa, and Bixby.

### 4.3 Interaction scenario

The interaction scenario involved the robot positioned in the middle of the room, facing participants who stood behind a table with a microphone to record their responses. Participants entered the experiment room at their scheduled times, were asked to take their positions, and then the experimenter delivered a monologue. Following this, participants completed a pre-questionnaire before signaling their readiness to begin the interaction. The robot introduced itself as EMAH, a student companion designed to engage in casual conversations and knowledge about RRLab. The background story varied across four sessions: In the first session, the robot knew nothing about the participant. In the second session, the robot started the conversation by summarizing the previous conversation history, and the participant was allowed to refer to their previous conversation topic. The third session introduced a transition function, theme switching, that activates if a conversation theme lasts more than six turns, allowing the robot to introduce a new topic. In the fourth and final session, the robot acknowledged that it was the last interaction and inquired about the participant’s feelings. After the first transition, it asked a scripted question: “I just remembered that the lectures have started. How’s it going for you?” The interaction concluded with the robot thanking the participant for their cooperation.

### 4.4 Research materials

In the multi-session student-robot interaction study, the robot used is named EMAH system implemented on Ameca robot. The study employed a mixed-method experimental design, incorporating several measures to evaluate various aspects of the interaction experience. These measures included the self-assessment manikin (SAM) (Bynion and Feldner, 2020), bot usability scale (BUS) (Borsci et al., 2022), human-robot interaction evaluation scale (HRIES) (Spatola et al., 2021), the Engagement construct from the Empathic Robots for Long-term Interaction Scale (Leite et al., 2014), the rapport-expectation scale (RERS) (Nomura and Kanda, 2016), the Perceived Safety construct from the Godspeed Questionnaire (Bartneck et al., 2009), and a post-experimental interview.

The pre-interaction survey collected information about participant demographics, prior contact with robots, the SAM using a five-point Likert scale, and robot perception from HRIES using a seven-point Likert scale. After each interaction session, participants completed the SAM again, followed by the five-point Likert BUS questionnaire and a Godspeed construct, which assessed participants’ views on the robot’s role during the interaction. In the fourth session, participants also completed seven-point Likert HRIES and RERS items, followed by a short interview. During the interview, participants answered two reflective questions.

(1) What do you think could make the robot better in interactions?
(2) What do you think you could have done better in the interactions?

All questionnaire items and evaluation metrics used in this study are provided in the supplementary material. Additionally, the conversation history, including human utterances, robot responses with timestamps, detected emotions of both the robot and the participant, and the participant’s background from each session, was recorded as supporting data.

## 5 Results and analysis

Analyses were carried out using JASP software version 0.19.1.

### 5.1 Repeated exposure and changes in social perception

To explore RQ1, a repeated measures ANOVA (RM-ANOVA) was conducted to assess changes in constructs of sociability, agency (Spatola et al., 2021) (see Supplementary Table S1), and engagement (Leite et al., 2014) (see Supplementary Table S2) across four interaction sessions. Mauchly’s test of sphericity was not violated for any construct (p>0.05) , allowing interpretation of standard ANOVA results. Polynomial contrasts were included for exploratory purposes but should be interpreted cautiously due to the small sample size.

Engagement scores remained relatively stable across sessions, with the mean engagement scores being: Post-1st = 3.79, Post-2nd = 3.90, Post-3rd = 4.15, and Post-4th = 4.12. Although a slight increase was observed descriptively, the main effect of session was not significant $\left(F\left(3,36\right)=1.386,\;p=0.263,\eta^{2}=0.104\right)$ , indicating that repeated interactions did not meaningfully increase engagement over time. Post hoc comparisons confirmed that no session-to-session differences were statistically significant (all p>0.05 ), with the largest observed mean difference being between Post-1st and Post-3rd sessions (Mdif=-0.365,SE=0.219,p=0.723) . Polynomial contrast tests further showed no significant linear (p=0.194) , quadratic (p=0.533) , or cubic (p=0.384) trends, suggesting engagement remained relatively stable across sessions. Sociability ratings also showed minimal change. Mean values across sessions were: Pre-1st = 4.42, Pre-2nd = 4.67, Pre-3rd = 4.38, and Pre-4th = 4.40. For sociability, RM-ANOVA results were not significant $\left(F\left(3,36\right)=0.844,\;p=0.479,\eta^{2}=0.066\right)$ , indicating no substantial increase in perceived sociability over time. Post hoc analyses showed no significant session-to-session differences (p>0.05) , though means fluctuated slightly. Polynomial contrasts for sociability were also non-significant, confirming the absence of reliable trends. For agency, mean scores across sessions were: Pre-1st = 5.17, Pre-2nd = 5.33, Pre-3rd = 5.15, and Pre-4th = 5.06. No significant main effect of session was observed $\left(F\left(3,36\right)=0.511,\;p=0.677,\eta^{2}=0.041\right)$ . Post hoc pairwise comparisons revealed no significant changes between sessions (p>0.05) , and polynomial contrast tests were non-significant across all trends (linear: p=0.454 , quadratic: p=0.334 , cubic: p=0.614 ).

Although none of the constructs demonstrated statistically significant change across the 4 HRI sessions, descriptive gender-based differences were noted. Males perceived a slight increase in sociability $\left(\Delta \tilde{X}=5\to 5.5\right)$ , while females reported improved sociability $\left(\Delta \tilde{X}=4\to 5\right)$ and reduced disturbance $\left(\Delta \tilde{X}=4\to 3\right)$ but lower agency $\left(\Delta \tilde{X}=6\to 5\right)$ , leaning towards prior findings that gender influences robotic perceptions (Spatola et al., 2021).

### 5.2 Prior robot contact and rapport development

To explore RQ2, a linear regression analysis was conducted to examine whether prior robot contact (“I have contact with social robots at university.“) predicted rapport with EMAH, measured as togetherness and partner perception (Nomura and Kanda, 2016) (see Supplementary Table S3).

Shapiro-Wilk tests confirmed that rapport variables were normally distributed (p>0.05) , validating the use of parametric tests, and VIF values were 1.000, indicating no multicollinearity. Pearson’s correlations showed weak positive relationships between prior robot contact and rapport constructs (expectations for togetherness: r=0.333,p=0.133 ; expectations as a conversation partner: r=0.366,p=0.109 ). However, these correlations did not reach statistical significance (p>0.05) , suggesting only a marginal association between prior robot contact and how participants perceived EMAH as a social partner. Descriptive statistics revealed moderate perceived rapport overall. For togetherness perception, scores ranged from 1.85 to 6.00 with a mean of 4.08 (SD = 1.22); for partner perception, scores ranged from 2.18 to 5.45 with a mean of 3.98 (SD = 1.05).

For the expectations for togetherness, the linear regression model explained 11.1% of the variance ( $R^{2}=0.111$ , adjusted $R^{2}=0.030$ ), but the regression was not statistically significant, F(1,11)=1.375,p=0.266 . The coefficient for prior robot contact was β=0.334,SE=0.285,t(11)=1.173,p=0.266 , 95%CI[−0.293,0.961] , indicating no strong predictive effect. For the expectations as a conversation partner, the model explained 13.4% of the variance ( $R^{2}=0.134$ , adjusted $R^{2}=0.056$ ), but again, the regression was not significant, F(1,11)=1.706,p=0.218 . The coefficient for prior robot contact was β=0.317,SE=0.243,t(11)=1.306,p=0.218 , 95%CI[−0.217,0.851] , suggesting only a weak, non-significant relationship. Effect sizes (Cohen’s $f^{2}$ ) for both models were small $\left(f^{2}<0.15\right)$ , reinforcing the low predictive value of prior robot contact on rapport outcomes. Residual diagnostics confirmed no major violations of linearity or homoscedasticity.

### 5.3 Familiarity and perceived disturbance

To explore RQ3, an RM-ANOVA was conducted to test whether the construct of perceived disturbance (Spatola et al., 2021) (see Supplementary Table S1) decreased over repeated interactions with EMAH administered in pre-surveys (referred to as Pre-1st, Pre-2nd, Pre-3rd, and Pre-4th). Shapiro-Wilk tests confirmed normality ( p>0.05 for all sessions). Mauchly’s test of sphericity was not violated (W=0.399,p=0.081) . Polynomial contrasts were included for exploratory purposes but should be interpreted cautiously due to the small sample size.

Descriptive statistics showed stable disturbance ratings: Pre-1st (M = 3.85, SD = 0.81), Pre-2nd (M = 3.85, SD = 0.79), Pre-3rd (M = 3.64, SD = 0.75), and Pre-4th (M = 3.62, SD = 0.72). The main effect of session was non-significant, $F\left(3,36\right)=0.425,\;p=0.736,\eta^{2}=0.034$ , indicating no meaningful reduction in perceived disturbance. Post-hoc comparisons revealed no significant pairwise differences (all p>0.05 ). Polynomial contrasts further confirmed a lack of linear (p=0.243) , quadratic (p=0.973) , or cubic (p=0.451) trends. These findings suggest no habituation effect in reducing perceptions of creepiness or uncanny feelings toward EMAH over repeated exposure.

By contrast, perceived animacy increased across sessions with a strong effect size $\left(\eta^{2}=0.473,p<0.001\right)$ . The mean scores were: Pre-1st (M = 3.08, SD = 0.70), Pre-2nd (M = 3.27, SD = 0.66), Pre-3rd (M = 4.38, SD = 0.62), and Pre-4th (M = 4.85, SD = 0.51). Post-hoc analyses revealed a significant increase in perceived animacy between Pre-1st and Pre-3rd sessions (p=0.003) and a small but significant increase between Pre-3rd and Pre-4th sessions (p=0.048) . Polynomial contrasts indicated a significant linear (p=0.002) , quadratic (p=0.020) , and cubic (p=0.004) trend, suggesting gradual improvement in the perception of EMAH’s lifelike qualities.

### 5.4 Expectations and willingness to use EMAH

To explore RQ4, a generalized linear model (GLM) with a Bernoulli distribution was conducted to examine whether the expectation of EMAH as a companion (Nomura and Kanda, 2016) (see Supplementary Table S3) predicts willingness to use EMAH as a university friend (“If EMAH is made specifically as a university student friend (companion), will you use it? (yes/no)”.). Preliminary analyses indicated that expectation togetherness and expectation partner were highly correlated (r=0.772,p<.001) , suggesting conceptual overlap. Shapiro-Wilk normality tests showed no significant deviations from normality for expectation togetherness (p=.860) and expectation partner (p=.467) , supporting their inclusion in the model. Descriptive statistics for these constructs are reported in Subsection 5.2.

Including the rapport construct of expectation togetherness significantly improved model fit over the null model (X2=4.463,p=.035) , but the predictor itself was not statistically significant (β=1.818,SE=1.177,p=.122) , with a wide confidence interval 95%CI[0.108,5.449] ). A separate GLM model, including expectation partner, also showed improved model fit (X2=6.413,p=.011) , indicating a stronger association. However, expectation partner did not significantly predict willingness to use EMAH (β=3.770,SE=3.220,p=.242) , with an odds ratio of 43.372, but a large confidence interval spanning −2.542 to 10.081, indicating instability in estimates. Due to severe multicollinearity (VIF=7.730 to 12.650) , expectation togetherness and expectation partner were tested in separate models to ensure stable estimation.

### 5.5 Perceived quality of SHR and LLM content

The Bot Usability Scale (BUS) (Borsci et al., 2022) (see Supplementary Table S2) assessed the LLM-powered SHR’s performance and content quality across four interaction sessions. An RM-ANOVA revealed a significant improvement in the SHR’s performance over time $\left(F\left(3,36\right)=3.353,\;p=0.029,\eta^{2}=0.218\right)$ , suggesting participants perceived the system as increasingly effective in handling conversations. In contrast, content quality ratings showed no significant changes across sessions $\left(F\left(3,36\right)=1.417,\;p=0.254,\eta^{2}=0.106\right)$ , with session 2 receiving the lowest median ratings, indicating probable inconsistencies in the relevance of content discussed. Mauchly’s test of sphericity was not violated for any construct (p>0.05) , allowing interpretation of standard ANOVA results.

Post hoc comparisons for SHR’s performance revealed a significant decline from the first to the third session (Mdif=-0.523,p=0.061) , with a moderate effect size (Cohen’s d = −0.583). However, no significant differences were observed between other sessions, indicating variability in perception but no consistent improvement or decline over time. Additionally, participants initially rated the SHR’s performance as neutral (Mode/Median=3) in the first two sessions, with improvements to four in later sessions, suggesting perceived functionality enhancements over time. In contrast, content quality dropped in 2nd session (Mode/Median=2) , indicating reduced relevance, before recovering to a neutral rating in later sessions.

### 5.6 Effect of human-robot interaction on mood

To assess whether participants interacting with an LLM-powered robot would show improvements in mood across four sessions, Wilcoxon signed-rank tests were conducted on the Pleasure, Arousal, and Dominance dimensions from the SAM non-verbal pictorial questionnaire (see Supplementary Figure S1), with pre- and post-session ratings. Data for the tests were not normally distributed, as confirmed by the Shapiro-Wilk test (all p<0.05 ); thus, non-parametric tests were used. The Wilcoxon signed-rank tests showed no significant differences in Pleasure, Arousal, or Dominance across the sessions. Specifically, no significant pre-post changes were observed for Pleasure, Arousal, or Dominance (e.g., Pleasure, Session 1: W=11.500,p=0.194 ; Session 2: W=20.000,p=0.395 ; Session 3: W=5.500,p=0.883 ; Session 4: W=6.000,p=0.187) .

Although not statistically significant p>0.05 ), descriptive trends emerged across the sessions. Pleasure showed a subtle but consistent rise, especially in Sessions 1 ( Δ = 0.308) and 3 ( Δ = 0.308). Arousal had a slight increase in Session 1 ( Δ = 0.384) and Session 3 ( Δ = 0.230) but showed no change in Session 2 ( Δ = 0.000). Dominance showed a rise in Sessions 1 ( Δ = 0.231), 2 ( Δ = 0.384), and 3 ( Δ = 0.307), with a slight decrease in Session 4 ( Δ = −0.154).

### 5.7 Experiment observation and content analysis

The system’s operation and human behavior were observed in real time during the multi-turn interaction sessions. Sentences containing background information, conversation history, and the timestamps of received utterances or system-generated responses were transcribed for further analysis. Several system limitations were identified through real-time observation. First, the response generation speed significantly slowed when utilizing the LLM + RAG approach (ranging from 6.0 to 15.0 s). Second, when dealing with complex or accented English speech, the automatic speech recognition (ASR) module often produced inaccurate transcriptions, particularly during user identification. Additionally, the clarity and structure of participants’ responses strongly influenced the quality of the conversation flow; more structured user input led to more engaging interactions.

The conversation data across four sessions revealed notable trends. Participants’ disclosure of personal information decreased between the first and third sessions but increased again in the fourth session. Similarly, conversation length and duration followed a decreasing trend up to the third session, with a partial recovery in the fourth. Turn-taking remained relatively consistent except for a notable drop in the third session, suggesting potential fatigue or decreased engagement. These patterns are detailed in Table 6. A Spearman’s correlation analysis was conducted to examine the relationship between perceived system quality, as measured by the BUS (Borsci et al., 2022), and conversational dynamics. A significant negative correlation between engagement and mean user turn-taking (p=0.046) suggested that as the robot became more engaging, participants tended to hold longer speaking turns. Conversation duration was positively correlated with the number of turn-takings (p=0.020) and showed a strong positive correlation with the amount of self-disclosure (p=0.002) .

**TABLE 6:** Mean values of conversational components across four sessions.

Observation component	Session 1	Session 2	Session 3	Session 4
Conv. duration (minutes)	19.986	18.496	12.256	15.526
Conv. length (words)	10.645	9.903	8.848	9.313
User turn-taking	30.615	30.538	24.231	29.231
Count of personal information detected	11.426	10.692	8.846	11.923

Post-session interviews further clarified participant perspectives on interaction quality. Overall, participants expressed enjoyment but highlighted several system limitations. Some noted that the robot would “insist on discussing the same topic” or “talk about things I was not aware of”, indicating issues with topic management and memory recall. Others observed that EMAH “sometimes looked around the room instead of keeping eye contact” and displayed “random expressions that did not match the conversation”. Regarding response quality, participants pointed out that “speech recognition errors made the answers confusing” and that responses sometimes felt “like talking to a four or five-year-old child, which is acceptable but needs improvement”. Suggested improvements included diversifying topic selection, enhancing memory and personalization, making facial expressions more natural, and reducing response times to better match human conversational pacing. Participants also reflected on their own role, suggesting that “preparing topics beforehand”, “responding in longer sentences”, and “being patient with response delays” could contribute to smoother, more natural interactions. These insights emphasize that both technological enhancements and user adaptation are critical for improving long-term human-robot interaction experiences.

## 6 Discussion and conclusion

This study explored how open-domain, multi-session interaction with an LLM-powered SHR affects user perceptions and willingness to adopt it as a social companion. Across four HRI sessions, we examined sociability, agency, engagement, rapport development, disturbance, animacy, expectations, and willingness to adopt the robot. Although they lacked strong statistical support, several minor trends emerged, offering valuable insights into long-term open-domain HRI using small, fine-tuned LLMs.

First, repeated interaction with EMAH showed that perceptions of sociability, agency, and engagement remained largely stable across sessions. Although high engagement was maintained through most of the study, it slightly declined during the final session. This suggests that while EMAH initially captured users’ attention, sustaining engagement over longer periods remains a challenge. Such declines may reflect user fatigue, novelty loss, or technical inconsistencies, as suggested by prior work on long-term HRI (Steinfeld et al., 2009; Smedegaard, 2019; Babel et al., 2021). Researchers have recommended personalization through tailored content as a means to counteract this well-documented HRI phenomenon (Nasir et al., 2021). Participant interviews reinforced this, with several users suggesting that EMAH should diversify topics, elaborate more naturally during discussions, and improve memory recall to make conversations feel less repetitive and more engaging.

Second, prior contact with robots did not significantly influence the development of rapport with EMAH. Although users with more robot experience reported slightly higher rapport descriptively, the effect was not statistically significant. This leans towards prior findings that mere exposure to robots does not guarantee deeper social bonding (Rosén et al., 2024).

Third, increasing familiarity through repeated sessions did not reduce perceptions of disturbance toward EMAH. However, animacy ratings increased, implying that familiarity made EMAH seem more lifelike without alleviating discomfort. This matches findings from HRI research showing that highly anthropomorphic designs often expose system limitations rather than conceal them (Berns and Ashok, 2024). Consistently, interview feedback in our study highlighted technical shortcomings, particularly in speech recognition and response timing. Participants noted that inaccurate transcriptions and long response delays disrupted conversational flow and made interactions feel less natural. These observations suggest that users may recalibrate their expectations (Rosén et al., 2022), viewing robots not as fully human-like agents, but rather as “scarecrows,” that are partially capable entities whose imperfections are anticipated and accepted (Williams et al., 2024).

Fourth, while higher expectations correlated with positive perceptions of sociability and engagement, they did not predict users’ willingness to adopt EMAH as a companion. Users can enjoy interacting with a robot without necessarily wanting a deeper relationship or repeated use, especially when subtle technical limitations persist (Kim C. Y. et al., 2024).

Another notable trend was the improvement in EMAH’s perceived functionality. Although initial ratings of the robot’s conversational ability were neutral, improvements were observed during the third and fourth sessions. This aligns with previous findings that familiarity can boost user confidence in system capabilities, promoting perceptions of reliability over time (Schneider and Kummert, 2021). However, the dip in functionality ratings between the first and third sessions suggests temporary inconsistencies in system performance. Similarly, the drop in conversation length and turn-taking during the third session likely reflected technical disruptions and participant fatigue. The recovery seen in the fourth session suggests that user engagement remained resilient, possibly supported by anticipation of completing the study (Oertel et al., 2020). This aligns with earlier findings that repeated interaction with social robots can lead to increased comfort and deeper disclosure over time (Laban et al., 2024).

At this point, we acknowledge that external factors likely influenced participant perceptions of the adapted open-domain LLM deployed on SHR EMAH. Speech recognition challenges, particularly with users’ accented English, and slow response generation likely affected user perceptions. It is important to note that response time ratings improved across sessions, suggesting some adaptation to EMAH’s Text-to-Speech pacing (Powell et al., 2022). Nonetheless, real-time observations confirmed that technical fragility contributed to fluctuations in participant ratings. Users appeared sensitive to inconsistencies in conversational quality, confirming earlier findings that real-world system flaws significantly shape perceptions (Berns and Ashok, 2024).

Overall, while the study found that SHR EMAH could sustain open-domain multi-turn interactions across multiple sessions, it also highlighted areas for future improvement. Future work will focus on enhancing the robot’s responsiveness, reducing inconsistencies in accented speech detection, and exploring ways to maintain engagement across longer-term interactions. Additionally, increasing the user sample size and diversity of participants could provide more robust insights into how different groups perceive and interact with the robot. This work contributes to the growing research on LLM-powered humanoid robots and their social perception in educational settings, offering insights into using customized LLMs in real-time, long-term HRI.

## Data Availability

The raw data supporting the conclusions of this article will be made available by the authors, without undue reservation.

## Footnotes

1
https://www.merphi.se/downloads/

2
https://youtu.be/mSKFp41aYyk

3
https://github.com/mauliana/Multi_Session-HRI

4
https://www.acapela-group.com/

5
https://pypi.org/project/alt-profanity-check/

6 Source: https://huggingface.co/datasets/samsum

7
https://huggingface.co/sentence-transformers/all-MiniLM-L6-v2

8
https://huggingface.co/hkunlp/instructor-large
