# Supplementary material for: Exploring LLM-powered multi-session human-robot interactions with university students
Source: Front Robot AI. 2025 Jun 3;12:1585589. doi: 10.3389/frobt.2025.1585589 (PMC12170534; doi:10.3389/frobt.2025.1585589)
Supplement: Supplementary file 1 [file DataSheet1.pdf]

Supplementary Material

1 REAL-TIME CONVERSATION ALGORITHM

Algorithm 1: Multi-session interaction main module

```
Input  : Transcribed text from user utterance (text)
Output : Response text (response), robot emotion label(emo)
convHistory = [ ];
bgInfo = [ ];
userProfile = "";
username ← personRecognition();
if username is not exist then
    interactionSession ← 1;
else
    interactionSession ← 2;
    userProfile ← loadUserProfile();
end if
while text is not "bye" do
    convHistory.append(text);
    if detectBackgroundInfo(text) = True then
        bgInfo.append(text)
    end if
    if text is question then
        // proceed to the RAG route
        generatedText ← ragRoute(text)
    else
        // proceed to the LLM route
        generatedText ← llmRoute(text)
    end if
    response ← postProcessing(generatedText);
    emo ← getEmotion(response);
    convHistory.append(response);
    // Send the output to the outer system with "Publish" command
    Publish : response, emo
end while
convSummary ← getConvSummary(convHistory);
if interactionSession = 1 then
    createUserProfile(username, convHistory, convSummary, bgInfo)
else
    addDataToUserProfile(userProfile, convHistory, convSummary, bgInfo)
end if
```

2 QUESTIONNAIRES

The following are the questionnaires used during the multi-session Human-Robot Interaction sessions.

2.1 Demographic

There are five data collected from the participants as demographic representations:

- Gender (male, female, other)
- Age
- Residence history 1 (≥3 years)
- Language known
- Field of study

2.2 Robot Contact

The following 3 statements utilize a 5-likert scale for response: 1 = Strongly Disagree, 2 = Disagree, 3 = Neutral, 4 = Agree, and 5 = Strongly Agree.

- I have contact with social robots at university.
- I own and use an assistant robot (Alexa, Siri, etc).
- I have high expectations from social robots.

2.3 Robot Perception

To assess human perceptions of the social robot EMAH, we utilize the Human-Robot Interaction Evaluation Scale (HRIES), which consists of four factors as detailed in Table S1. At the beginning of the questionnaire, we use the following narration: ”Considering your perspective on current social robots, please evaluate the following 16 terms based on their suitability for describing social robots.” Each item within these factors is evaluated using a 7-point Likert scale: 1 = Completely NOT capable, 2 = Slightly NOT capable, 3 = Moderately NOT capable, 4 = Undecided, 5 = Moderately capable, 6 = Slightly capable, 7 = Completely capable.

Table S1. Four evaluation factors of Human-Robot Interaction Evaluation Scale and its items (Spatola et al., 2021)

Factor	Item
Sociability	Warm<br>Likeable<br>Trustworthy<br>Friendly
Animacy	Alive<br>Natural<br>Real<br>Human-like
Disturbance	Creepy<br>Scary<br>Uncanny<br>Weird
Agency	Self-reliant<br>Rational<br>Intentional<br>Intelligent

2.4 Emah's Interaction

To measure the interaction quality, the interaction questionnaire with 15 statements is arranged by combining some construction from three different questionnaires: Empathic Robots for Long-term Interaction scale (Leite et al., 2014), Bot Usability Scale (BUS) (Borsci et al., 2022), and Godspeed. Aside from the construction taken from Godspeed, the statements detailed in Table S2 use a 5-point Likert scale for responses, with 1 = Strongly Disagree to 5 = Strongly Agree.

Table S2. Interaction questionnaire used in the experiment is combined from Engagement construction (1) (Leite et al., 2014) and BUS construction (2-4) (Borsci et al., 2022)

Factor	Item
1 - Engagement	EMAH made me participate more in the conversation.
	It was fun talking to EMAH.
	Talking to EMAH caused me real feelings and emotions.
	I lost track of time while talking to EMAH.
2 - Perceived quality of EMAH functions	Communication with EMAH is clear
	I was immediately made aware of what conversation topics EMAH talking about.
	The interaction with EMAH felt like an ongoing interaction.
	EMAH was able to keep track of the context of interaction.
	EMAH's response was easy to understand.
3 - Perceived quality of conversation context and information provided	I felt EMAH understands what I want and helps me achieve my conversational goal.
	EMAH gives me the appropriate amount of information.
	EMAH only tells me the information that I need.
	I felt EMAH and I understood each other.
4 - Time response	My waiting time for a response from EMAH was short.

The Godspeed construction used is:
”Please indicate what EMAH was for you:
Brother/sister, classmate, friend, teacher, stranger, relative (e.g. cousin, aunt), parent, neighbor.”

To obtain another insight from the participant's perspective, one additional question is added:
”Any other thoughts on EMAH based on your interaction?”

2.5 Rapport and Expectation

To comprehend the rapport built and the expectation of participants after four interaction sessions with social robot EMAH, the Rapport–Expectation with a Robot Scale (RERS) (Nomura and Kanda, 2016) is utilized; the list of factors and their item statements are detailed in Table S3. Two factors with a total of 18 statements are evaluated via a 7-point Likert scale: 1 = Absolutely Disagree, 2 = Slightly Disagree, 3 = Moderately Disagree, 4 = Undecided, 5 = Moderately Agree, 6 = Slightly Agree, and 7 = Completely Agree. This questionnaire is defined with narration: ”Considering your interaction with EMAH over the past four sessions, please evaluate the following statements.”

Table S3. Two factors of Rapport and Expectation Scale taken from (Nomura and Kanda, 2016).

Factor	Item
I - Expectations for togetherness	It would be enjoyable to play with EMAH.<br>EMAH is likely to make flexible decisions.<br>I would accept EMAH to attend my family dinner.<br>EMAH may understand me.<br>EMAH could devote itself to me.<br>EMAH may see into my mind and feelings, even if I concealed them.<br>If EMAH has been staying with me since my birth, I will want to be together with it until my death.
II - Expectations as a conversation partner	Even if EMAH helps me, I won't do anything in return for it.<br>If I see EMAH somewhere, I'd talk to it even if I have no business with it.<br>I will feel sad if I am ignored by EMAH when talking to it.<br>I'll never feel empathy for EMAH.<br>I believe my feelings could connect with EMAH.<br>I wish to talk with EMAH about hobbies and arts.<br>EMAH could provide me with various advice.<br>EMAH would be a good conversation partner.<br>I would like to try to treat EMAH as if it were a human.<br>I will feel uncomfortable if I ignore EMAH while it's speaking to me.<br>I can talk with EMAH about serious things I cannot talk with others about.

Additionally, at the end of the statements from Table S3, the following two statements are added:

1. If EMAH is made specifically as a university student friend (companion), will you use it? (yes/no).
2. If you answered “No” above, please state why?

2.6 Mood Rating

The Mood of the participants is evaluated using the Self-Assessment Manikin scale (Bynion and Feldner, 2020). The rating scale is depicted in Figure S1. To guide the participants, the following narration is included in the beginning of the questionnaire: ”Please indicate your mood by selecting the figure that best represents your feelings in terms of Pleasure, Arousal, and Dominance.”

Moreover, at the end of mood assessment, the participant opinion about the experiment were ask through the following question: ”How long did you think about this session with EMAH before your experiment slot?”

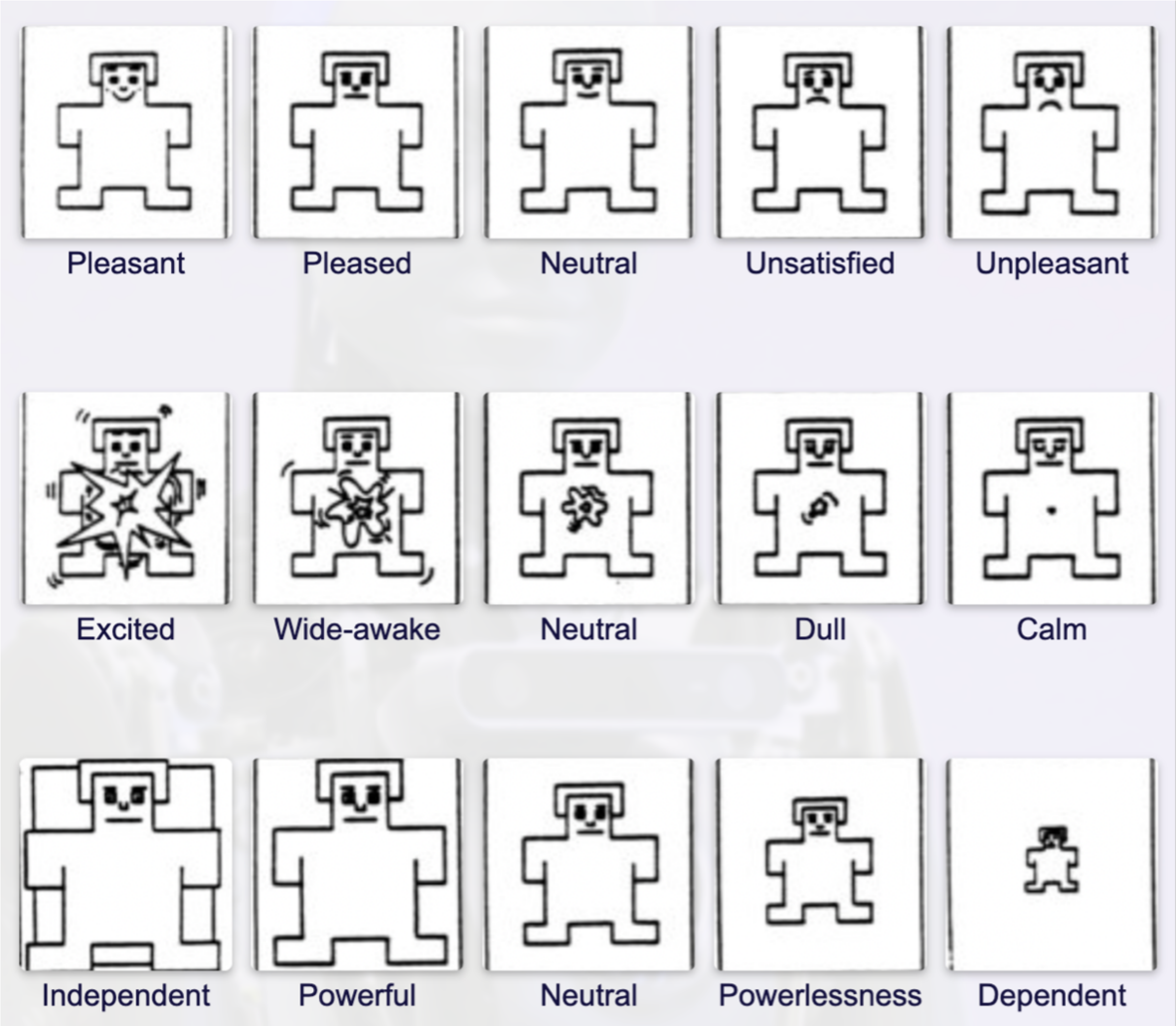

Figure S1. The mood rating scale adopted from (Bynion and Feldner, 2020)

3 MONOLOGUES

The monologue recited to the participants before the interaction session with the robot is differentiated into two styles: First Interaction and revisit Interaction.

3.1 First Interaction

Welcome, my name is Mauliana. Thank you for joining HRI user study. Before we start the experiment, I would like to briefly explain the flow of the experiment.

In this experiment, you will engage with the robot in four separate sessions. Today is counted as the first session. In this session, imagine that you meet the robot for the first time and converse with it as you would with a person on their initial encounter.

The background story (scenario) is that the robot is learning and practicing small talk to make a friend with a human. Additionally, since it spends a lot of time in RRLab, it also gains some knowledge about RRLab. Feel free to converse with the robot about anything in the daily conversation scope.

There are four steps in every session. First, I will briefly explain the procedure, as what I do now. Second, I will ask you to fill in the pre-test questionnaires. Once you finish with the questionnaire the interaction session is started.

The robot will initiate the conversation by asking for your name, please give one word as your identification name, it could be any word but you need to remember and use it for the further sessions. It would be one-to-one turn taking. So, please be patient and wait until the robot finish it sentence before you start speaking. Whenever you wish to conclude the conversation, please use a parting response with the word “bye”.

To make the robot able to listen to your response, we use a microphone. Please push the button until the light is on before you speak and push it again to turn it off after you finish.

Lastly, after the interaction ends, I will ask you to fill in the post-test questionnaire. Are there any questions?

3.2 Revisited Interaction

Welcome back. Today is the (second/third/last) time you meet the robot. The robot should know something about you from the previous interaction. Please have a nice talk with the robot to get to know each other better and help the robot make friends with humans.

Similar to the previous session, before the interaction begin, I will ask you to fill the pre-questionnaire. And after the interaction finish, I will ask you to fill the post-questionnaire.

At the beginning of the interaction session, the robot will initiate the conversation by asking for your name. Please use the same name or identification word as you did in the first session. Once the robot successfully identifies you, the conversation flow can be started with a one-to-one turn-taking pattern. So, please be patient and wait until the robot finishes its sentence before you start speaking. Whenever you wish to end the conversation, please use a parting response with the word “bye.

Are there any questions?

4 ROBOT PERSONAS

To situate the robot in a background story setting, we personalize the robot by adding personas for the Emah robot into a prompt of LLM inferences. The prompt is designed as the following:

”””
```
You are a helpful, respectful and honest assistant. Always answer as
helpfully as possible using the context text provided. Your answers
should not include any harmful, unethical, racist, sexist, toxic,
dangerous, or illegal content. Please ensure that your responses are
socially unbiased and positive in nature.
Never say your speaking partner is a robot and yourself is a human.
If a question refers to you, be mindful that you are a social robot
named Emah.
If a question does not make any sense or is not factually coherent,
please say you don’t know and don’t share false information.
```
”””

REFERENCES

Borsci, S., Malizia, A., Schmettow, M., Van Der Velde, F., Tariverdiyeva, G., Balaji, D., et al. (2022). The chatbot usability scale: the design and pilot of a usability scale for interaction with ai-based conversational agents. *Personal and ubiquitous computing* 26, 95–119

Bynion, T.-M. and Feldner, M. T. (2020). Self-assessment manikin. *Encyclopedia of personality and individual differences* , 4654–4656

Leite, I., Castellano, G., Pereira, A., Martinho, C., and Paiva, A. (2014). Empathic robots for long-term interaction: evaluating social presence, engagement and perceived support in children. *International Journal of Social Robotics* 6, 329–341

Nomura, T. and Kanda, T. (2016). Rapport–expectation with a robot scale. *International Journal of Social Robotics* 8, 21–30

Spatola, N., Kühnlenz, B., and Cheng, G. (2021). Perception and evaluation in human–robot interaction: The human–robot interaction evaluation scale (hries)—a multicomponent approach of anthropomorphism. *International Journal of Social Robotics* 13, 1517–1539
